# *Enterobacter cloacae *infection of an expanded polytetrafluoroethylene femoral-popliteal bypass graft: a case report

**DOI:** 10.1186/1752-1947-4-131

**Published:** 2010-05-09

**Authors:** Ian Musil, Vanessa Jensen, Jolyon Schilling, Boyd Ashdown, Tyler Kent

**Affiliations:** 1 College of Medicine, University of Arizona, 1501 N Campbell Avenue, Tucson AZ 85724, USA; 2 Department of Surgery, University of Arizona, 1501 N. Campbell Avenue, PO Box 245066 Tucson, Arizona 85724-5066, USA; 3 Tucson Hospitals Medical Education Program, 1501 N. Campbell Avenue, PO Box 245066, Tucson, Arizona 85724-5066, USA

## Abstract

**Introduction:**

*Enterobacter cloacae *infections are common among burn victims, immunocompromised patients, and patients with malignancy. Most commonly these infections are manifested as nosocomial urinary tract or pulmonary infections. Nosocomial outbreaks have also been associated with colonization of certain surgical equipment and operative cleaning solutions. Infections of an aortobifemoral prosthesis, an aortic graft, and arteriovenous fistulae are noted in the literature. To our knowledge, this is the first isolated account of an *E. cloacae *infection of a femoral-popliteal expanded polytetrafluoroethylene bypass graft.

**Case presentation:**

A 68-year-old Caucasian man presented with fever and rest pain in the right lower extremity five months after the placement of a vascular expanded polytetrafluoroethylene graft for femoral-popliteal bypass. Computed tomography angiography demonstrated peri-graft fluid that was aspirated percutaneously with image guidance and cultured to reveal *E. cloacae*. The graft was revised and then removed. The patient completed a six-week course of ceftazidime and is currently without signs of infection. There were no other reports of *E. cloacae *graft infections in any patients receiving treatment in the same surgical suite within a month of this report.

**Conclusion:**

Isolated cases of *E. cloacae *infection of surgical bypass grafts are rare (unique in this setting). Clinicians should have a high index of suspicion for device contamination in such cases and should consider testing for possible microbial reservoirs. Graft removal is required due to the formation of biofilm and the recent emergence of Enterobacteriaceae producing extended-spectrum beta-lactamase in community acquired infections.

## Introduction

*Enterobacter cloacae *infections are seen commonly in burn victims, immunocompromised patients, and patients with malignancy [1]. The urinary and pulmonary systems are the organ systems most commonly colonized in these patients. *E. cloacae *bacteremia can also occur depending on the extent of immunocompromise.

Outbreaks of coloacae infections are recorded in a number of hospital settings. Sporadic cases of *E. cloacae *infections have been linked to contaminated admixed intravenous fluids, total parenteral nutrition solutions, enteral feedings, infant formula, cardioplegic solution, and blood products [2-4]. Another potential reservoir for nosocomial bacteremia is the heparin flush solution used to irrigate certain intravascular devices continually. This fluid had been implicated as a reservoir for outbreaks of device-associated bacteremia in several instances [5].

Less commonly, outbreaks are linked to the colonization of devices such as long-term tunneled hemodialysis catheters. Buxton and colleagues reported an epidemic of *E. cloacae *infections that was associated with disposable transducer domes. During their initial setup the chambers and domes were contaminated by the hands of hospital personal who had handled contaminated transducer heads [6]. West and colleagues also reported *E. cloacae *sepsis resulting from transducer dome cracks. In this study, disposable transducer domes were being re-sterilized with resultant cracks in the dome membrane holding bacteria. Case series also document the colonization of entire operative suites that require extensive decontamination [4].

## Case presentation

A 68-year-old male presents to a rural clinic with a two-week history of claudication and a one-day history of rest pain and fever. His past medical history was relevant for lower extremity arterial insufficiency and claudication in his right leg for which he underwent a right femoral-popliteal above the knee bypass using a 6mm expanded polytetrafluoroethylene (ePTFE) graft in May 2007. The graft was revised a month later in July 2007 when surveillance ultrasound revealed an occlusion. Intra-operative findings at that presentation included extensive calcific atherosclerotic disease and aggressive intimal hyperplasia in the popliteal artery. A small seroma around the distal anastomosis was discovered and evacuated at that time. There was no evidence of graft infection after thrombectomy and revision. Our patient was started on coumadin to maintain graft patency. His initial recovery was unremarkable.

Four months later, our patient began to experience claudication for two weeks and then developed rest pain and fever. He did not display any tissue loss or sensory changes. On physical exam, our patient's distal incision was warm and slightly indurated. His temperature was 38.5°C. Pedal pulses were 2+ in the left extremity and could not be palpated on the right.

**Figure 1:**
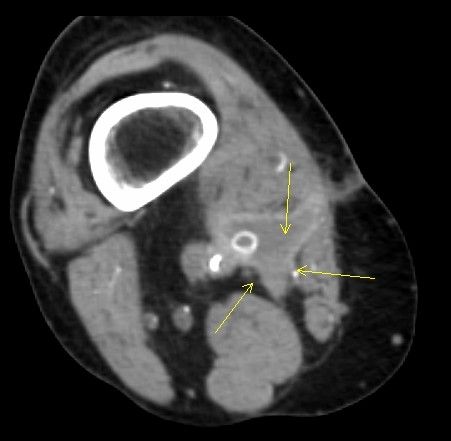
**CT angiogram of a small seroma in the right popliteal fossa taken prior to seroma biopsy and removal of the femoral-popliteal ePTFE bypass graft**. Arrows point to the location of the seroma.

**Figure 2:**
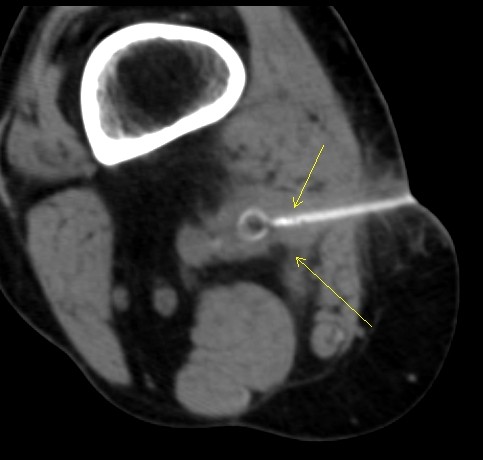
**CT image used in correlation with CT-guided drainage of the abscess created by graft infection**. Cultures of aspirated material revealed colonies of *E. cloacae*. Arrows point to needle biopsy.

Computed tomography angiography (CTA) of our patient (Figure 1) revealed a peri-graft fluid collection in the popliteal fossa of the right lower extremity and an occluded femoral-popliteal graft. Our patient's white blood cell count was 9000 cells with a 73% neutrophilia and his sedimentation rate was 64 Westergren units (mm/h). A CT-guided aspiration of the peri-graft fluid (Figure 2) was cultured and revealed *E. cloacae *with sensitivity to extended-spectrum cephalosporins, including cefepime, cefpirome, and cefclidine. At that time, our patient was started on a third generation cephalosporin, 1g intravenous ceftazidime every eight hours.

Six hours later, the patient underwent removal of the graft. Endarterectomy with saphenous vein patch angioplasty was performed on the common femoral artery for severe atherosclerotic stenosis. Collaterals from the deep femoral artery were relied upon to perfuse the limb. Our patient completed a six-week course of ceftazidime, currently uses the limb without discomfort, and is without signs of infection.

## Discussion

Synthetic peripheral bypass vascular grafts carry a high morbidity [7]. The risk of complications is as high as 50% in some studies. These risks include hemorrhage, limb ischemia, amputation, revision, or infection [7,8]. Rates for peripheral lower extremity prosthetic graft infection are as high as 12% [7]. Risk factors for graft infection are similar to those for wound infection and include obesity, diabetes, cancer and other immunocompromised states, as well as the use of immunosuppressive therapies. Our patient had a history of lung cancer in remission, but was without other risk factors.

The most common pathogen in graft infection is *Staphylococcus aureus*, which is responsible for 35% of graft infections. Gram-negative rods account for another 20% of vascular graft infections. Other common organisms in these infections include coagulase negative Staphylococcus, *Streptococcus milleri, Enterococcus faecium, Escherichia coli, Bacteroides fragilis, Candida albicans *and *Pseudomonus aeruginosa*. Polymicrobial colonization is commonly seen in immunocompromised patients. Grafts that require groin incisions have the greatest risk for infection, possibly due to contamination by bowel flora at the time of implantation [9].

Nosocomial *E. cloacae *infections are not entirely uncommon. Their isolated occurrence among vascular prosthetic grafts is rare, however. There are accounts of *E. cloacae *infection of an aortobifemoral bypass prosthesis and reports of *E. cloacae *infection in other types of vascular grafts (including an aortic graft), but, to our knowledge, this is the first account of an *E. cloacae *infection in a femoral-popliteal ePTFE bypass graft [10,11]. ePTFE is a woven form of PTFE, the same polymer used in Teflon^© ^(DuPont). Research demonstrates this material is particularly prone to infection and biofilm formation relative to autologous tissues [12].

From the clinical perspective, biofilms are a major problem, since these structures display greatly increased resistance to physical and chemical insults. Crucially, biofilms are resistant to antibiotic treatment, making them particularly difficult to eliminate from patients and contaminated surgical equipment [13]. Several case reports, as well as larger studies from Canada, France, Israel, Spain, Italy and the UK indicate that infections caused by extended spectrum Beta-lactamase (ESBL) Enterobacteriaceae is an emerging problem in outpatient settings [15]. Therefore, graft removal should always be considered.

Prosthetic vascular graft infections commonly occur without specific symptoms [14]. Some authors suggest surveillance in the absence of clinical symptoms with the use of indium scan to identify infection. Clinical symptoms arose in this patient before surveillance could be conducted. When signs and symptoms are present, diagnoses of graft infection is still difficult and must often rely on clinical basis alone as blood cultures are often negative [16]. Most authors suggest the use of CTA as the most appropriate study to identify infection when clinical suspicion is high [17]. Peri-graft fluid, ectopic air, abnormal tissue planes or soft tissue swelling, and pseudoaneurysm formation are all signs of graft infection on CT. Non-specific findings include fever, leukocytosis with left shift, and elevated inflammation markers (erythrocyte sedimentation rate, ESR; C-reactive protein, CRP). Absence of other signs of graft infection should prompt a thorough workup for other causes. CTA was used to identify the nidus of infection in our patient.

Management requires resection of the infected graft, arterial repair or bypass with autologous tissue, and long-term antibiotics [18]. Placement of a new prosthetic graft should be avoided in the presence of acute infection. Adjuvant techniques of endarterectomy or percutaneous revascularization may be helpful. The importance of standard hospital and operating room hygiene measures (disinfection of hands) must not be underestimated. These measures are paramount to the control and prevention of infection. When an isolated surgical case of *E. cloacae *occurs, clinicians should have a high index of suspicion for device contamination, consider testing for possible microbial reservoirs, and also consider documenting the case in the literature [14].

## Conclusions

Currently, our patient experiences mild claudication, but no rest pain. He has no evidence of infection following the removal of his ePTFE graft, endarterectomy of the common femoral artery, saphenous vein patch, and intravenous ceftazidime. Additionally, there are no records of *E. cloacae *infection of any other patient at this hospital within a month of original graft placement. This makes widespread contamination of surgical equipment unlikely.

## Consent

Written informed consent was obtained from the patient for publication of this case report and any accompanying images. A copy of the written consent is available for review by the Editor-in-Chief of this journal.

## Competing interests

The authors declare that they have no competing interests.

## Authors' contributions

VJ analyzed and interpreted the patient data regarding the vascular graft infection and was a major contributor in writing the manuscript. JS removed the graft, performed common femoral artery, and performed a saphenous vein patch of the common femoral artery. JS was also a major contributor in writing the manuscript. IM analyzed the patient's medical history and all patient labs and imaging studies, as well as contacting the patient for consent. IM was the major contributor to writing the manuscript. All authors read and approved the final manuscript.
